# *In vitro* susceptibility pattern of *Rhodococcus equi* isolated from patients to antimicrobials recommended exclusively to humans, to domestic animals and to both

**DOI:** 10.1590/S1678-9946202567003

**Published:** 2025-02-03

**Authors:** Nícolas Garcia Ribeiro, Paulo da Silva, Patrick Júnior de Lima Paz, Marcelo Fagali Arabe, Fernando Paganini Listoni, Evandro Paganini Listoni, Letícia Colin Panegossi, Márcio Garcia Ribeiro

**Affiliations:** 1 Fundação Educacional do Município de Assis, Faculdade de Medicina, Assis, São Paulo, Brazil; 2 Instituto Adolfo Lutz, Ribeirão Preto, São Paulo, Brazil; 3 Universidade Estadual Paulista, Faculdade de Medicina Veterinária e Zootecnia, Departamento de Produção Animal e Medicina Veterinária Preventiva, Botucatu, São Paulo, Brazil

**Keywords:** Human rhodococcosis, Multidrug-resistant bacteria, Molecular diagnosis, MALDI-TOF MS

## Abstract

*Rhodococcus equi* is an opportunistic soil-borne bacterium that is eliminated in feces of multi-host animals. An increase in multidrug-resistant *R. equi* isolates has been reported in humans and domestic animals, and it has been hypothesized that the treatment of *R. equi* in foals could increase the selective pressure on multidrug-resistant isolates and favor human infections by resistant isolates. We investigated the *in vitro* antimicrobial susceptibility/resistance of 41 *R. equi* strains from humans, which were isolated from patients with pulmonary signs, using 19 antimicrobials from 10 distinct classes, recommended exclusively to humans, recommended exclusively to domestic animals and used in both. All isolates were subjected to mass spectrometry and identified as *R. equi.* Among the antimicrobials used exclusively in humans, tigecycline and vancomycin showed 100% efficacy. Amikacin, amoxicillin/clavulanic acid, imipenem, levofloxacin, clarithromycin, rifampin, ciprofloxacin, and gentamicin, used in both humans and animals, revealed high efficacy (97–100%). Conversely, a higher frequency of isolates was resistant to penicillin (87.8%) and trimethoprim/sulfamethoxazole (43.9%), which are used in both humans and animals. Among the antimicrobials used only in animals, isolates were resistant to florfenicol (46.4%), ceftiofur (17.1%), and enrofloxacin (2.5%). Multidrug resistance was observed in 34% of isolates. The identification of drug-resistant *R. equi* isolated from humans used exclusively in animals is circumstantial evidence of the pathogen transmission from domestic animals to humans. This study contributes to the molecular identification of *Rhodococcus* species from humans and to the epidemiological vigilance of the multidrug-resistant isolates.

## INTRODUCTION

*Rhodococcus equi* are opportunistic bacteria in nature belonging to the Actinomycetes class, Mycobacteriales order, which include other pathogenic agents relevant to humans and animals, e.g*.*, *Mycobacteria, Nocardia,* and *Corynebacteria* (National Center for Biotechnology Information – NCBI, 2024). More than 40 species of the *Rhodococcus* genus are recognized^1^, although *R. equi* has been described as a primary pathogen to humans, domestic animals, and wildlife^2^.

*R. equi* is a soil-borne pathogen, commonly found in the soil, feces, and organic matter of livestock farms (horses, cattle, pigs), and is eliminated in feces of different domestic animals^3^.

The pathogen is characterized by facultative intracellular infections in phagocytic cells (neutrophils and macrophages) and the development of suppurative-to-pyogranulomatous lesions^4^. The virulence of *R. equi* has been attributed to capsule and mycolic acid in the cell wall, exoenzymes and more recently to plasmids associated with virulence (pVAP), which favor the intracellular permanence of the pathogen in phagocytic cells^5^ and, consequently, chronic infections^6^. To date, three host-adapted virulent types are known (pVAPA, pVAPB and pVAPN)^7^, which are eliminated mainly in feces of horses (foals), pigs, and cattle/small ruminants, respectively^4^. Nonetheless, humans are susceptible to infection by the three types of pVAPs^2,4,6,8,9^, demonstrating the pathogen’s zoonotic nature^4,8,10^.

Among humans, *R. equi* infections have been increasingly identified and considered an emerging disease in some countries^8,10^. *R. equi* infections are most commonly reported in individuals with or coinfected by debilitant or immunosuppressed diseases, particularly those living with acquired immunodeficiency syndrome (AIDS), caused by human immunodeficiency virus (HIV)^6,8,10-12^, and is considered a health concern both worldwide^8^, and in Brazil^13,14^. Nonetheless, the pathogen is also able to infect immunocompetent humans^6,12^.

Inhaling aerosols from contaminated environments on livestock farms is considered the main route of human infection^12,15^; although, in recent years, studies suggest that the ingestion of contaminated (feces or lymph node contents) or undercooked beef and pork may represent an alternative transmission route, explaining the disease in human patients who are not in contact with livestock and their environments^8,14^.

Cavitary pneumonia is the most frequent and severe clinical manifestation of human rhodococcosis. Moreover, diarrhea, organ abscesses, progressive weight loss, lymphadenitis, nephritis, peritonitis, arthritis, osteomyelitis, and meningitis represent extrapulmonary signs also reported in human *R. equi* infections^12,15^.

The routine diagnosis of rhodococcosis in humans is based on clinical and epidemiological data, microbiological culture and imaging, and cytological and histopathological examinations^12,15^. Microbiological culture is mainly performed using respiratory secretions, blood, and abscess contents, particularly as a differential of tuberculosis, due to the similarity between mycobacteria and *R. equi* as primary agents of pulmonary infections in humans^16,17^. In addition, molecular methods such as PCR, sequencing^8,14^, and matrix-assisted laser desorption ionization time-of-flight mass spectrometry (MALDI-TOF MS)^18^ have enabled the confirmation of *R. equi* species and the identification of its plasmid virulence pattern^8,14,16^.

Rifamycins (rifampin), glycopeptides (vancomycin), carbapenems (imipenem), beta-lactam derivatives (cephalosporins, amoxicillin/clavulanic acid), macrolides (erythromycin, clarithromycin), fluoroquinolones (ciprofloxacin), and sulfonamides are considered the main groups/antimicrobials for treating human rhodococcosis^12,15,17,19-21^. However, an increase in the multidrug resistance of *R. equi* isolates found in humans and domestic animals (e.g*.*, rifampin, erythromycin, and sulfonamides) has been reported worldwide and considered a human health issue^15,17,22-24^. In this context, human infections may be acquired through direct contact with livestock animals or their farm environment^10,20^, including foals. Young horses are animals in which *R. equi* infections are usually treated with antimicrobials similar to those used for human rhodococcosis, (i.e*.*, rifampin, erythromycin, azithromycin, and clarithromycin)^3,25,26^, and it has been recently hypothesized that humans could be infected by multidrug-resistant isolates from foals treated for rhodococcosis^10,20^.

Considering the increasing number of human rhodococcosis cases, the emergence of multidrug resistance in *R. equi* from humans and animals, and the risks of human infection by multidrug-resistant isolates from livestock, we investigated the *in vitro* antimicrobial susceptibility/resistance of *R. equi* from humans, which were isolated from patients with pulmonary signs that resemble tuberculosis, using antimicrobials recommended exclusively to humans, exclusively to domestic animals, and to both, in which the species-identification of isolates was performed using mass spectrometry.

## MATERIALS AND METHODS

### Ethics

This study was conducted under the Scientific Committee of Adolfo Lutz Institute (CLR-RP-VI-IAL) of Ribeirao Preto, SP (protocol Nº 52-P/2023), and the Ethics Committee on Animal Use (CEUA) guidelines of the School of Veterinary Medicine and Animal Science (FMVZ), Sao Paulo University (UNESP), Botucatu, SP (protocol Nº 123/2015), Brazil.

### Rhodococcus equi isolates and human patients

A convenience sampling of 41 *R. equi* strains isolated from diseased humans showing clinical signs of pneumonia, whose primary supposed diagnosis was pulmonary tuberculosis, was used. The isolates were provided by the bacteriology laboratories from the Adolfo Lutz Institute, Ribeirao Preto, SP (Centro de Laboratorio Regional de Ribeirao Preto - VI - Instituto Adolfo Lutz), and the School of Veterinary Medicine and Animal Science, Sao Paulo State University (UNESP), Botucatu, SP, Brazil. No epidemiological data from patients included in the study were used for further analysis to maintain their complete anonymity.

### Microbiological culture

*R. equi* strains were isolated from pulmonary secretions in sheep blood agar media (5%) (Oxoid^***®***^, Sao Paulo, Brazil), and aerobically incubated for three days at 37 ***°*** C, in addition to being cultured aerobically in Lowenstein-Jensen media (Oxoid^***®***^, Sao Paulo, Brazil) for 90 days at 37 ***°*** C.

White-to-gray, circular, mucoid, coalescent colonies, with 1–2 mm diameter after 48–72 h of culture, common traits of *Rhodococcus* species, were subjected to traditional phenotyping methods, including the morphology of the organisms to Gram staining (coccoid to pleomorphic Gram-positive bacteria) and a positive CAMP test using *Staphylococcus aureus* ^17,27^. Isolates were kept in microtubes frozen at −80 ***°*** C (15% glycerol) or in Lignières media, at 25 ***°*** C.

### Mass spectrometry

The confirmation of *R. equi* at the species level was performed via matrix-assisted laser desorption/ionization time-of-flight mass spectrometry (MALDI-TOF MS, Bruker and Daltonics^TM^, Bremen, Germany) with a 337-nm laser. The spectra were analyzed between 2,000–20,000 m/z using the FlexControl software (version 3.3, Bruker Daltonics, Bremen, Germany). Identification of the microorganisms at the genus and species levels was considered ≥1.7 and ≥2.0, respectively^28^.

### In vitro antimicrobial susceptibility testing

All isolates were subjected to an *in vitro* antimicrobial susceptibility test (disk diffusion method) based on the Clinical Laboratory Standards Institute (CLSI)^29,30^, using 19 antimicrobials from 10 classes: 1) drugs used exclusively in humans (tigecycline, vancomycin); 2) drugs used in both humans and animals (amikacin, amoxicillin/clavulanic acid, azithromycin, ceftriaxone, ciprofloxacin, clarithromycin, erythromycin, gentamicin, imipenem, levofloxacin, penicillin, rifampicin, sulfamethoxazole/trimethoprim); and 3) drugs used only in animals (ceftiofur, enrofloxacin, florfenicol, marbofloxacin)^29-31^. Due to the lack of specific standardization of interpretations of disk diffusion test for the *in vitro* susceptibility of *Rhodococcus* species, the criteria on *Staphylococcus* species was used^17,32-34^. Isolates that exhibited resistance to >3 different classes (groups) of antimicrobials were considered multiresistant^35^.

## RESULTS

### Mass spectrometry

All 41 isolates were identified as *R. equi* based on mass spectrometry via the MALDI-TOF MS method.

### In vitro susceptibility pattern

Table 1 summarizes the antimicrobial patterns of the 41 *R. equi* isolates. Among antimicrobials used exclusively in humans, tigecycline and vancomycin had 100% efficacy. The amikacin, amoxycillin/clavulanic acid, imipenem, levofloxacin, clarithromycin, and rifampin used in humans and animals were also effective in all (100%) isolates, while the ciprofloxacin and gentamicin also revealed high efficacy (>97%).

**Table 1:** *In vitro* antimicrobial profiles (disk diffusion test) of 41 *Rhodococcus equi* isolated from humans.

		Susceptibility		
Groups	Antimicrobials	S (%)	I (%)	R (%)
Aminoglycosides	Amikacin gentamicin	41/41 (100) 40/41 (97.5)	-- 1/41 (2.5)	-- --
Beta-lactam and derivatives	amoxicillin/clav. acid	41/41 (100)	--	--
	ceftiofur	29/41 (70.7)	5/41 (12.2)	7/41 (17.1)
	ceftriaxone	33/41 (80.5)	2/41 (4.9)	6/41 (14.6)
	imipenem	41/41 (100)	--	--
	penicillin	5/41 (12.2)	--	36/41 (87.8)
Fluoroquinolones	ciprofloxacin	40/41 (97.5)	1/41 (2.5)	--
	enrofloxacin	33/41 (80.5)	7/41 (17)	1/41 (2.5)
	levofloxacin	41/41 (100)	--	--
	marbofloxacin	41/41 (100)	--	--
Glycilcyclines	tigecycline	41/41 (100)	--	--
Glycopeptide	vancomycin	41/41 (100)	--	--
Macrolides	azithromycin	21/41 (51.2)	13/41 (31.7)	7/41 (17.1)
	clarithromycin	41/41 (100)	--	--
	erythromycin	33/41 (80.5)	8/41 (19.5)	--
Phenicols	florfenicol	13/41 (31.7)	9/41 (21.9)	19/41 (46.4)
Rifamycins	rifampin	41/41 (100)	--	--
Sulfonamides	trimethoprim/sulfa	20/41 (48.8)	3/41 (7.3)	18/41 (43.9)

S = susceptible; I = intermediate, R = resistant; amoxicillin/clav. acid = amoxicillin/clavulanic acid; trimethoprim/sulfa = trimethoprim/sulfamethoxazole. ◼ Antimicrobials recommended exclusively for humans; □ Antimicrobials recommended exclusively for animals; ◻ Antimicrobials used in both humans and animals

A higher frequency of isolates was resistant to penicillin (87.8%) and trimethoprim/sulfamethoxazole (43.9%), which are used in both humans and animals. Moreover, among antimicrobials used only in animals, isolates were resistant to florfenicol (46.4%), ceftiofur (17.1%), and enrofloxacin (2.5%). Multidrug resistance was observed in 34% (14/41) of isolates. Among the multidrug-resistant isolates, 21.9% (9/41) and 12.1% (5/41) were resistant to three and four classes of antimicrobials, respectively.

## DISCUSSION

In this study, 41 *R. equi* strains isolated from pulmonary infections of humans and identified by mass spectrometry were subjected to 19 antimicrobials from 10 classes, including those used exclusively in humans, those used exclusively in animals, and those used in both. All isolates were susceptible to tigecycline and vancomycin, which are recommended exclusively to humans, while some drugs used in both humans and animals exhibited high efficacy (>97%). Nonetheless, high multidrug resistance (34%) of isolates was observed, including florfenicol, ceftiofur, and enrofloxacin, which are used exclusively for animal therapy. These findings reinforce concerns regarding the worldwide increase in multidrug-resistant *R. equi* isolates of human origin. In addition, the presence of *R. equi* isolated from humans resistant to drugs used exclusively in animals is circumstantial evidence of the transmission of isolates from domestic animals to humans, particularly from foals, in which therapy for clinical rhodococcosis is common, highlighting the need for rational use of antimicrobials in therapeutic approaches for *R. equi* infections in domestic animals.

More than 40 *Rhodococcus* species have been reported^1^, although *R. equi* has been recognized as the major species of the pathogen in humans, domestic animals, and wildlife. However, sporadic human infections caused by other *Rhodococcus* species have been previously reported, e.g*.*, *R. rhodochrous*, *R. erythropolis*, and *R. fascians* ^2^.

In the current study, *R. equi* was identified in all 41 isolates and confirmed at the species level based on mass spectrometry, via the MALDI-TOF MS technique. In addition to the routine diagnosis of *R. equi* from human origin via traditional phenotyping methods^17^, molecular techniques such as PCR, sequencing^8,14^, and mass spectrometry^18^ have enabled the confirmation of *Rhodococcus* species isolated from humans and identification of the plasmid virulence patterns of isolates for epidemiological purposes^8,14,16^. A study investigated 154 *Rhodococcus* strains isolated from human patients with HIV/AIDS, pneumonic foals, and isolates from the environment, and MALDI-TOF MS was a fast and reliable technique^36^ for the diagnosis of *R. equi* ^18^, indicating mass spectrometry is a valuable method to diagnose *R. equi* infections in humans.

The first identification of *R. equi* (formerly *Corynebacterium equi*) as a human pathogen was reported in a man from the USA with lung abscesses and impaired immune response, with prolonged corticoid therapy due to chronic hepatitis^37^, whereas the first human case related to a patient living with HIV/AIDS was reported in a man with a lung abscess and persistent bacteremia^38^. In Brazil, *R. equi* infection was first reported in a patient with pulmonary signs coinfected with HIV^39^. To date, *R. equi* infections in humans have increased, and the disease is considered to be emerging in some countries^8,10^, related mainly in debilitated or immunosuppressed patients, particularly in humans living with HIV/AIDS^6,8,10-12^, which is a human concern worldwide^6,8,23,24^, including Brazil^13,14,17^. However, *R. equi* can also infect immunocompetent humans^6,12^.

Although extrapulmonary infections have been reported, pulmonary abscesses and cavitary pneumonia are the prevalent clinical signs of rhodococcosis in humans^12,15,16^, which is consistent with the findings of this study, where 41 strains were isolated from the pulmonary samples of diseased patients who resembled clinical signs of pulmonary tuberculosis, reinforcing pneumonia as the major clinical sign of rhodococcosis in humans. In addition, our study highlights that *R. equi* could be considered a differential diagnosis of tuberculosis, particularly in Brazil^33^, among patients who are in contact with livestock, their farm environment, or individuals who consume fresh or undercooked meat from cattle and pigs^6,8,9,13^.

Rifamycins, glycopeptides, carbapenems, beta-lactam derivatives, macrolides, fluoroquinolones, and sulfonamides are considered the main groups of antimicrobials used to treat human rhodococcosis^12,15,17,19-21^. In fact, among the 41 *R. equi* strains included in this study and subjected to an *in vitro* antimicrobial susceptibility pattern (disk diffusion method), vancomycin (glycopeptide) and tigecycline (glycilcycline), two drugs used exclusively for human therapy, showed 100% efficacy. In addition, amikacin, gentamicin (aminoglycosides), amoxycillin/clavulanic acid (beta-lactam), imipenem (carbapenem), levofloxacin, and ciprofloxacin (fluroquinolones), clarithromycin (macrolide), and rifampin (rifamycin), which are used to treat humans and animals, also showed high efficacy (between 97-100%), being good options for therapeutic approaches for *R. equi* infections in humans.

Conversely, an increase in the resistance of *R. equi* isolated from diseased humans and domestic animals (especially to rifampin, erythromycin, and sulfonamides) has been reported in various countries, including Brazil^17^, and is considered a human health issue^15,21-24^. In this study, *R. equi* isolates revealed higher resistance to penicillin (87.8%) and trimethoprim/sulfamethoxazole (43.9%), as well as moderate resistance to azithromycin (17.1%) and ceftriaxone (14.6%), which are antimicrobials used for therapy in humans and animals. Furthermore, 34% of isolates exhibited multidrug resistance to three (21.9%) or four (12.1%) antimicrobial classes, respectively. This finding highlights the increased number of multidrug-resistant *R. equi* isolates recovered from diseased humans, and reinforces the need for vigilance of multidrug resistance of the pathogen isolated from human infections. Additionally, given the increase of multidrug-resistant *R. equi* isolates worldwide, therapeutic approaches should be initiated based on previous *in vitro* susceptibility tests^4^.

Human infections caused by *R. equi* may be acquired directly through contact with diseased livestock animals or their environment^10,20^, although the risk of transmission from companion animals to humans remains unclear^3^. Dogs and cats with rhodococcosis have been sporadically treated with antimicrobials^3,40^. Nonetheless, young horses (foals up to six months of age) represent an animal category in which treatment for *R. equi* infections is usually performed, using antimicrobials similar to those used for human rhodococcosis, e.g*.*, rifampin, erythromycin, azithromycin, and clarithromycin^3,24-26^. It has been recently hypothesized that humans could be infected by multidrug-resistant *R. equi* isolates from diseased foals subjected to treatment^10,20^. Among the antimicrobials tested in 41 human isolates of *R. equi*, which are used exclusively in animals, a high resistance of isolates to florfenicol was observed (46.4%), in addition to a minor resistance of strains to ceftiofur (17.1%) and enrofloxacin (2.5%). This finding is circumstantial evidence of the transmission of multidrug-resistant *R. equi* isolates from domestic animals to humans, particularly from foals, where selection pressure on resistant isolates likely occurs because antimicrobial is commonly used in this animal. Nonetheless, further studies could focus on genetic similarities between *R. equi* strains isolated from humans and domestic animals to confirm the risk of transmission of multidrug-resistant isolates from domestic animals to humans.

Some limitations in this study are convenience sampling, lack of standardization of the disk diffusion method to *in vitro* antimicrobial susceptibility tests of actinomycetes (including *Rhodococcus* species), and lack of sequencing approaches that investigate genetic similarities between *R. equi* strains isolated from humans and livestock or companion animals.

## CONCLUSION

A total of 41 *R. equi* strains isolated from humans with pulmonary infections were identified via mass spectrometry, reinforcing this species as the most common in human infections. All *R. equi* were subjected to 19 antimicrobials from ten different classes, using drugs exclusive to human therapy, drugs exclusively used in animals, and drugs used in both humans and animals. All isolates were susceptible to tigecycline and vancomycin, recommended exclusively for humans. Some drugs used for both humans and animals, belonging to the aminoglycosides, beta-lactams, carbapenems, fluoroquinolones, macrolides, and rifamycins classes, revealed high efficacy (>97%), indicating these antimicrobials also represent valuable options for disease therapy. In contrast, high multidrug resistance (34%) of isolates was observed, reinforcing the worldwide issue regarding the increase in multidrug-resistant *R. equi* isolates of human origin. The presence of *R. equi* isolated from humans resistant to drugs exclusive for the therapy of domestic animals is circumstantial evidence of the transmission of *R. equi* isolates from domestic animals to humans, highlighting the need for rational use of antimicrobials in therapeutic approaches of rhodococcosis in domestic animals.
